# Mutations in Two Aphid-Regulated *β*-1,3-Glucanase Genes by CRISPR/Cas9 Do Not Increase Barley Resistance to *Rhopalosiphum padi* L

**DOI:** 10.3389/fpls.2020.01043

**Published:** 2020-07-09

**Authors:** Sung-Yong Kim, Therese Bengtsson, Niklas Olsson, Vehbo Hot, Li-Hua Zhu, Inger Åhman

**Affiliations:** Department of Plant Breeding, Swedish University of Agricultural Sciences, Alnarp, Sweden

**Keywords:** *Hordeum vulgare* L., *β*-1,3-glucanase, CRISPR/Cas9, susceptibility, plant breeding, insect pest

## Abstract

Callose deposition is induced in plants by various stress factors such as when plants are attacked by herbivores and pathogens. In the case of aphids, callose plugging of aphid-damaged phloem sieve tubes is expected to reduce aphid access to the phloem sap, while aphid-induced upregulation of callose-degrading *β*-1,3-glucanase genes in the host plant might counteract this negative effect on aphid performance. We have tested this hypothesis with barley mutants in which one or both of two *β*-1,3-glucanase genes (1636 and 1639) have been mutated by CRISPR/Cas9 technique in cv. Golden Promise. These two genes were previously found to be upregulated by the cereal pest *Rhopalosiphum padi* L. in susceptible barley genotypes. Four 1636/1639 double mutant, three 1636 single mutant and two 1639 single mutant lines were tested for aphid resistance along with control lines. All mutant lines had single base insertions, causing frame shifts and premature stop codons. Three of the four double mutant lines showed significantly reduced *β*-1,3-glucanase activity, and bacterial flagellin-induction resulted in significantly more callose formation in the leaves of double mutant compared to control and single mutant lines. However, we found no effect of these modified plant traits on barley resistance to *R. padi*. Both genes were confirmed to be upregulated by *R. padi* in Golden Promise. The gene 1637 is another *β*-1,3-glucanase gene known to be upregulated by *R. padi* in barley and was here found to be higher expressed in a double mutant line when compared with a control line. If this can compensate for the general reduction of *β*-1,3-glucanase activity in the double mutants is difficult to discern since phloem concentrations of these proteins are unknown.

## Introduction

Aphids are phloem-feeding insects. Out of approximately 4,700 species in the Aphididae family, *ca* 100 are crop pests (Blackman and Eastop, 2007). They damage plants directly when feeding on phloem sap, but also indirectly by vectoring viral diseases. The most common aphid control method in field crops is to apply insecticides. To grow aphid-resistant cultivars is a control method that is more environmentally friendly, less risky for human health, less costly, and more easily applied. However, relatively few cultivars have been bred for resistance to their aphid pests so far. Among the examples are wheat and barley cultivars with resistance to one or both of *Schizaphis graminis* Rondani and *Diuraphis noxia* Kurdjumov (Mornhinweg et al., 2012; Tolmay et al., 2015; Mornhinweg et al., 2017), soybean cultivars resistant to *Aphis glycines* Matsumura (https://extension.umn.edu/soybean-variety-selection/aphid-resistant-soybean-varieties-minnesota; accessed on 11 November 2019), a lettuce cultivar resistant to *Nasonovia ribisnigri* (Mosley), and other aphid species (http://agris.fao.org/agris-search/search.do?recordID=CZ2000000225; accessed on 11 November 2019) and melon cultivars resistant to *Aphis gossypii* Glover (Boissot et al., 2016).

Plant resistance traits may confer both physiological (antibiosis) and behavioral (antixenosis) effects on the insect (Smith, 1989). Causes for insect resistance are thus more complicated to study than causes for disease resistance. However, compared to chewing herbivorous insects, aphids make only small mechanical damage to their hosts and aphids are relatively sedentary once they have accepted a plant to feed and reproduce on. These are factors that may explain why aphid/host interactions show many similarities with pathogen/host plant interactions. Classical genetic studies or mapping of aphid/plant interactions indicate that dominantly inherited resistance genes, *R* genes, in several cases take part in the resistance reaction (Dogimont et al., 2010). Two of three aphid-resistance genes that have been sequenced so far, *Mi-1* in tomato and *Vat* in melon, belong to the nucleotide-binding-site leucine-rich repeat (NBS-LRR) family of *R* genes (Rossi et al., 1998; Dogimont et al., 2014, respectively). Such genes are involved in plant recognition of certain effectors secreted by pathogens and pests. Early plant responses to the attack include rapid depolarization of the plasma transmembrane potential, a rise in cytosolic Ca^2+^, production of reactive oxygen species, and mitogen-activated protein kinase signaling. Subsequently, plant hormones interact with transcription factors involved in production of plant secondary metabolites, defense proteins, and other plant defense traits (Erb and Reymond, 2019). For example, when rice expressing the *R* gene *Bhp14* is infested with the phloem feeding planthopper *Nilaparvata lugens* Stål, the salicylic acid signaling pathway is upregulated, which in turn induces trypsin inhibitor production and callose deposition in phloem causing resistance to the planthopper (Du et al., 2009). However, experience with deployment of cultivars with single *R* gene resistance is that sooner or later it is overcome by other biotypes/races of the plant antagonist. This has indeed been the case with cereals resistant to the aphids *S. graminis* and *D. noxia* as well as soybean resistant to *A. glycines* and lettuce to *N. ribisnigri* (Dogimont et al., 2010; Jaouannet et al., 2014).

An alternative strategy to breed for resistance is to reduce plant susceptibility (Pavan et al., 2010; Åhman et al., 2019), rather than to, as in most of the examples mentioned above, introduce *R* genes. This approach is now facilitated by the novel genome editing method CRISPR/Cas9, which can be used to make a double DNA strand break (DSB) (Shan et al., 2013; Belhaj et al., 2015). The DSB is subsequently repaired by the native non-homologous end joining mechanism (NHEJ), which may erroneously add or delete one or a few nucleotides that may, in turn, result in loss of gene function. There are now several examples where this method has been tested and found useful for breeding disease resistant plants (Weeks et al., 2015; Mushtaq et al., 2019; Sedeek et al., 2019).

To exploit this method in breeding for resistance to aphids it is necessary to first identify candidate genes for susceptibility (Åhman et al., 2019). Here we have chosen to study *β*-1,3-glucanases which might have a role in reducing clogging of the phloem sieve elements by the *β*-1,3-glucan callose. Sieve tubes consist of modified plant cells connected *via* sieve plates. Phloem sap is transported through callose-lined pores in the sieve plates. Upon mechanical damage to the phloem, these pores are clogged firstly by expanding proteins and somewhat later by callose. These plant reactions are Ca^2+^-dependent (Van Bel and Will, 2016). However, when aphids puncture the sieve elements there are in most aphid/plant combinations just minor clogging of the sieve plates, allowing the aphid to feed from the same phloem tube for substantial time (Tjallingii, 2006). The reason may be that Ca^2+^-binding proteins in aphid-exuded saliva reduce build-up of protein and callose plugs (Will et al., 2013). An alternative or complementary explanation is that effectors in aphid saliva induce increased plant production of *β*-1,3-glucanases that can degrade the *β*-glucan callose (Giordanengo et al., 2010; Van Bel and Will, 2016). There are many *β*-1,3-glucanases with different functions at different stages of plant development, such as during microspore development, pollen germination, pollen tube extension, fruit development, ripening, and seed germination (Balasubramanian et al., 2012; Pirselova and Matusikova, 2013). Certain of the *β*-1,3-glucanases belong to the so called pathogenesis-related (PR) proteins (Van Loon and van Strien, 1999; Doxey et al., 2007) induced by pathogens and believed to either target fungal pathogens directly or to elicit other plant defense mechanisms *via* release of glucan as a damage signal (Balasubramanian et al., 2012). In general, disease resistant plant genotypes show faster accumulation of glucanases than the susceptible ones (Balasubramanian et al., 2012). However, in several studies comparing resistant and susceptible genotypes of aphid-induced plants the relation is the reverse, as determined at the gene expression level. Certain *β*-1,3-glucanase transcripts are found to be more abundant in the susceptible than in the resistant genotypes, for example in the interactions between *S. graminum* and sorghum (Park et al., 2006), wheat (Reddy et al., 2013) and switchgrass (Koch et al., 2018), between *Acyrthosiphon pisum* Harris and barrel medic (Sun et al., 2018a), *Sipha flava* Forbes. and switchgrass (Koch et al., 2018), and *R. padi* and barley (Delp et al., 2009; Mehrabi et al., 2016). However, there are also examples where *β*-1,3-glucanase transcripts or proteins are more abundant in the resistant genotype (Van der Westhuizen et al., 1998; Forslund et al., 2000; Van der Westhuizen et al., 2002; Botha et al., 2014; Sun et al., 2018b).

In the present study we have developed and characterized barley (*Hordeum vulgare* L.) mutant lines, in which two *β*-1,3-glucanase genes (contigs 1636 and 1639) were mutated in the cultivar Golden Promise (GP) using CRISPR/Cas9 technique. These are two of the three glucanase genes commonly found to be upregulated by *R. padi* in barley germplasm (Delp et al., 2009; Saheed et al., 2009; Mehrabi et al., 2016). Our hypothesis is that mutations in one or both of these two genes will hamper the aphid’s ability to, indirectly *via* the host plant, reduce callose deposition in the sieve tubes of barley. The reduced breakdown of callose is in turn anticipated to reduce aphid access to the phloem sap and thereby negatively affect aphid growth and potentially influence aphid host preference as well. This would be a novel way of breeding for resistance to *R. padi*, which is a serious pest of barley in temperate regions worldwide (Blackman and Eastop, 2007).

## Materials and Methods

### Development and Characterization of Mutant Lines

#### Cloning of the Genes 1636 and 1639

Two *β*-1,3-glucanase genes in GP were PCR-amplified using genomic DNA and cDNA as templates and the primers were designed according to the sequence with accession no X67099 in National Center for Biotechnology Infromation (NCBI) for 1636 and the sequence with accession no AK248899 in NCBI for 1639. The genomic DNA was extracted from young leaf tissues using Thermo Scientific Gene Jet plant genomic DNA purification mini Kit (USA) and RNA using Qiagen RNA extraction Kit (USA) according to the manufacturers’ instructions. cDNA synthesis was performed using SuperScript III first-strand synthesis systems according to the manufacturer’s instructions (Thermo Fisher, USA). The PCR conditions were 98˚C for 3 min, 30 cycles of 98˚C for 10 s, 61˚C for 1 min, 72˚C for 1 min, and final extension of 72˚C for 10 min. The PCR primers used are shown in **Table 1**. The PCR products were sequenced by GATC biotech (Germany) and compared with the sequences with the accession numbers mentioned above.

**Table 1:** Primers and sgRNA sequences used in this study.

Name	Sequence
**For cloning of the target genes**	
1636-For	5’-ATGGCGAGGAAAGGTGTAGACGTCGCAGTGGC-3’
1636-Rev	5’-CTAGAAAGTAATGGCGTAGGCCGGTGACATAT-3’
1639-For	5’-ATGCAAATACATACGCACCAAGTTATGATAAG-3’
1639-Rev	5’-TCAGAAAGTAATGGAGTAGGCCGGCGACTTGT-3’
**For CRISPR target sequences (sgRNA) of 1636 and 1639 genes**	
sgRNA-1	5’-**G**TCGGCGTCTGCAACGGCGT-3’^a^
sgRNA-2	5’-**G**TGCGGATCTACGAGCCGGA-3’^a^
sgRNA-3	5’-**G**ACTCCATCGGCGTCTGCAA-3’^a^
sgRNA-4	5’- **G**CTCACGGCGCTCAGCGGCA-3’^a^
**For PCR of the target genes for high-resolution fragment analysis (HRFA)**	
1636 HRFA For	5’-HEX-ATGGCGAGGAAAGGTGTAG-3’
1636 HRFA Rev	5’-GAGGAGACGTTGGCCTTTAC-3’
1639 HRFA For	5’-FAM-GATAAGATCGTCGATGGCGAAG-3’
1639 HRFA Rev	5’-CTTCGCGCCGGGCACCACCGTG-3’
**For sequencing of putative mutation lines**	
1636-For	5’-ATGGCGAGGAAAGGTGTAGACGTCGCAGTGGC-3’
1636-Rev	5’-CTAGAAAGTAATGGCGTAGGCCGGTGACATAT-3’
1639-For	5’-ATGCAAATACATACGCACCAAGTTATGATAAG-3’
1639-Rev	5’-ATGGCTGGGAGGATGGT-3’
**For PCR of the hygromycin resistance gene**	
Hyg-For	5’-GATGTAGGAGGGCGTGGATA-3’
Hyg-Rev	5’-GATGTTGGCGACCTCGTATT-3’

^a^G was added before the target sequences to facilitate the U6 promoter transcription.

#### Phylogenetic Analysis of Glucanase Genes and Localization of Glucanases

Apart from the two genes 1636 and 1639, there is another, 1637 (accession no AJ271367), which was previously shown to be induced by *R. padi* in various barley germplasm (Delp et al., 2009; Saheed et al., 2009; Mehrabi et al., 2016). In order to evaluate how similar the three genes are in GP, and also to compare them with other *β*-1,3-glucanase genes in the barley databases, we blasted these three genes against the genome sequence of cv. Morex at IPK’s barley blast server (http://webblast.ipk-gatersleben.de/barley_ibsc/) and made a phylogenetic analysis of all the putative *β*-1,3-glucanase genes using Phylogeny.fr (maximum likelihood with bootstraps) software. For predicted localization of proteins related to the glucanase genes, the sequences were uploaded to Bologna unified subcellular component annotator (BUSCA; http://busca.biocomp.unibo.it). BUSCA predicts subcellular localization using programs for signal and transit peptides (DeepSig and TPpred3), GPI-anchors (PredGPI), transmembrane domains (ENSEMBLE3.0 and BetAware), and for subcellular localization (BacelLo, MemLoci, and SChloro). Actual isoenzyme information and subcellular protein localization refer to information by Li et al. (1996) and UniProt.org (https://www.uniprot.org/).

#### CRISPR/Cas9 Vector Construction

The target sequences of the two *β*-1,3-glucanase genes were selected using the CRISPR-design tool CRISPR RGEN (http://www.rgenome.net). Two CRISPR vectors were constructed where the target sequences were expressed under the monocot-optimized rice U6 promoter. Each target sequence neighboring a 5’-NGG PAM was 19 bp with an additional G inserted to facilitate the U6 promoter-based transcription. Each vector harbors two target sequences and one of them targeted both genes whereas the other was gene specific. The target sequences in vector 1 were sgRNA-1 for targeting both genes and sgRNA-2 for targeting 1636, and in vector 2 were sgRNA-3 for targeting both genes and sgRNA-4 for targeting 1639 (**Table 1**).

Preparation of the CRISPR vectors and plant transformation were carried out at John Innes Centre (JIC), UK, as described by Lawrenson and Harwood (2019) and Hinchliffe and Harwood (2019).

#### Growth Conditions in the Biotron

The transgenic rooted plantlets from JIC were transferred from *in vitro* to soil in 1.5 L pots and grown in a growth chamber in the biotron at SLU, Alnarp. To accelerate the subsequent generation cycles, speed breeding conditions were adopted (Watson et al., 2018), namely 20 h photoperiod at 22˚C and 4 h dark at 20˚C and small pot size. Relative humidity was 80% and photosynthetically active radiation (PAR) was 500 μmol m^−2^s^−1^ at plant level using metal-halogen lamps.

#### Identification and Characterization of Mutant Lines

Genomic DNA was extracted from lyophilized and grinded young leaves. 500 µl lysis buffer (0,1 M Tris-HCl, 20 mM EDTA, 1,4 M NaCl, and 2% CTAB, pH 7,5) was added and samples were incubated at 52°C for 15 min. Following centrifugation at 2,000*g* for 15 min, 200 µl supernatant was transferred to Qiacube HT for DNA extraction using the QIAamp 96 DNA QIAcube HT Kit (Qiagen, Hilden, Germany). The DNA was used for PCR and subsequent high-resolution fragment analysis (HRFA) (Andersson et al., 2017) using the capillary electrophoresis-instrument Genetic Analyser 3500 (Thermo Fisher Scientific, Waltham, USA). In HRFA the size of a fluorescently labeled PCR product of a putative mutant was compared with an internal standard and the wild type PCR product. Gene-specific primers were designed with a fluorescent dye (Sigma-Aldrich, St. Louis, USA) attached to the 5’-end of the forward primers (**Table 1**). PCR was performed in a reaction containing 1X Phusion HF buffer, 0,2 mM dNTPs, 0,25 µM of each primer, 0,2 units of Phusion polymerase, 1 µl DNA extract, and water up to 10 µl (Thermo Fisher Scientific, Waltham, USA). The PCR conditions were 98˚C for 3 min, 35 cycles of 98˚C for 10 s, 61˚C for 10 s, 72˚C for 20 s, and final extension of 72˚C for 10 min. For sequencing, PCR products amplified with the non-labeled primers under the same PCR conditions were ligated into the pJET1.2/blunt vector, which was then transformed into *E. coli.* DH5α. The plasmid DNA was isolated using plasmid extraction kit from Thermo Fisher (USA). The primers were the same as for gene cloning except for the reverse primer for 1639 (**Table 1**). The PCR products were sequenced by GATC biotech (Germany). PCR of the hygromycin resistance gene was performed to screen for transgene-free plants using primers yielding a product of 450 bp analyzed by electrophoresis using 1.2% agarose gel (**Table 1**). PCR conditions were 98˚C for 3 min, 30 cycles of 98˚C for 10 s, 61˚C for 1 min, 72˚C for 1 min, and final extension of 72˚C for 10 min.

#### β-1,3-Glucanase Activity

The *β*-1,3-glucanase activity in the seedlings of the mutant lines along with the controls (see result section for the detailed information about the plant materials and number of replicates used) was assessed based on the dinitrosalicylic acid (DNS) method (Miller, 1959) using laminarin as a substrate (Sigma L-9634, USA). Proteins were extracted from the green parts of two weeks old seedlings. The seedlings were frozen in liquid nitrogen and ground with mortar and pestle in the protein extraction buffer from Agrisera (Sweden), supplemented with proteinase inhibitors (Roche, USA). Protein amount was quantified using the BCA protein assay kit from Thermo Fisher, USA. The protein extract (100 μl) was mixed with 100 μl of 2% (w/v) laminarin and incubated at 37˚C for 1 h. The reaction was terminated by adding 1 ml of 1% (v/v) staining DNS and boiling for 5 min. After cooling down at room temperature, the solution was diluted 1:20 in distilled water and the absorbance at 500 nm was measured by spectrophotometer. The *β*-1,3-glucanase activity was estimated as nmol of released reducing sugar (D-glucose) per hour per milligram of soluble protein.

#### Callose Visualization and Quantification

The second leaf from two weeks old seedlings was cut into 1 cm long pieces and then incubated with 1 µM Flg22 in 150 mM K_2_HPO_4_ buffer for 24 h at room temperature. Flg22 is a callose-inducing peptide derived from bacterial flagellin (Gomez-Gomez et al., 1999). Meanwhile, untreated leaf pieces were also incubated in the buffer. After treatment, the leaves were de-stained in 1:3 acetic acid/ethanol until transparency. After washing in 150 mM K_2_HPO_4_ for 30 min, the leaves were incubated with 0.01% aniline blue in 150 mM K_2_HPO_4_ solution for 2 h in dark. Thereafter, the leaves were preserved in 50% glycerol. Callose depositions were observed at 370/509 nm (aniline blue excitation/emission wave lengths) using a fluorescence microscope with DAPI filter (Leica DMLB, Germany). Amount of callose was quantified from the digital photos of Flg22-treated leaves as number of bright pixels using the Adobe software Photoshop’s “Record measurement” tool (cf. Luna et al., 2011).

### Aphid Tests

#### Aphid Rearing in the Greenhouse

Rearing was started with winged *R. padi* collected from the winter host *Prunus padus* L. in Saxtorp in 2018 and in Alnarp in 2019. The aphids were reared on oats (*Avena sativa* L.) in cages in a greenhouse chamber kept at minimum 18˚C and minimum 16 h light, natural light supplemented by 400W HQIE lamps.

#### Barley Plants for Aphid Tests

For all aphid tests, seeds were soaked in water on filter paper in a refrigerator (4–8^0^ C) for 3 d, thereafter germinated in the laboratory for 2 d. The seedlings were planted in Emmaljunga exklusiv Blom & Plantjord soil (Emmaljunga torvmull AB, Vittsjö, Sweden) in 10 cm-diameter plastic pots, placed for testing in greenhouse or biotron chambers. Young plants were used for aphid tests since *R. padi* performs best during seedling to stem elongation of barley (Leather & Dixon, 1981).

#### Aphid Regulation of β-1,3-Glucanase Gene Expression

**Figure 1:**
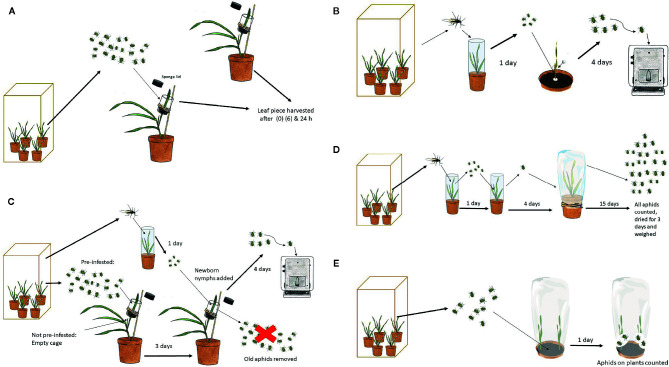
Procedures for tests involving *R. padi*. **(A)** Induction with aphids for analysis of plant *β*-1,3-glucanase regulation. **(B)** Aphid individual weight after growing on test plants. **(C)** Aphid individual weight after growing on test plants pre-treated with other aphids compared to not pre-treated. **(D)** Aphid population size and biomass on test plants. **(E)** Aphid host choice between a mutant and a control plant.

The expression levels of the two genes 1636 and 1639 were analyzed in GP by qRT-PCR to evaluate the aphid effects on gene expression (**Figure 1A**). Barley plants were grown in a climate-controlled growth chamber in the biotron, illuminated with PAR 200 μmol m^−2^s^−1^ at plant level using metal-halogen lamps 16 h per day, at a temperature of 20˚C and relative humidity 80%. The experiment was started 15 d after planting, in which 20 aphids were caged on the mid-section of the second leaf in the same type of cage as was used for the test of aphid individual growth after pre-infestation (see below). There were five replicates per treatment, each one including two plants from which the caged sections of the leaves were pooled. The treatments included control 1 (without cage) with leaf samples taken at time 0, control 2 (with cage) after 6 and 24 h and aphid-infested after 6 and 24 h. The leaf samples were immediately frozen in liquid nitrogen and stored at −80˚C for further use.

In order to test if the 1637 gene expression was differently induced in a double mutant in comparison to a non-mutated genotype segregated from the same transformation event, the induction experiment was also carried out with a 1636/1639 double mutant line and a control line, but the sampling was at 0 and 24 h only and with four replicates per treatment.

#### RNA Extraction and cDNA Synthesis

The frozen leaf samples were homogenized in a Retsch Mixer Mill MM400 (Retsch GmbH, Haan, Germany) for 1 min at 30 Hz. Total RNA was extracted using the RNeasy Mini Kit (Qiagen GmbH, Hilden, Germany) and the remaining genomic DNA was removed using RNase-free DNase I (Qiagen) following manufacturers’ instructions. RNA concentration and purity (260/280 nm >1.8) were checked using a ND-1000 NanoDrop (Wilmington, USA), and the integrity was analyzed by gel electrophoresis using E-Gel^®^ 1.2% (Life Technologies, Carlsbad, CA). The DNA-free RNA (0.6 µg) was then reverse-transcribed to cDNA using Superscript III reverse transcriptase enzyme (Life Technologies).

#### RT-qPCR Analysis

RT-qPCR was performed using a CFX96 Touch™ Real-Time PCR Detection System (Bio-Rad Laboratories, Inc., Hercules, CA, USA) for all three *β*-1,3-glucanase genes. In addition, two reference genes, 20S proteasome alpha subunit E (*SF427*) and heat shock protein (*HvHsp70*) were included for normalization. The PCR protocol and primer sequences were the same as previously published by Mehrabi et al. (2016). The data normalization was performed according to the method described by Vandesompele et al. (2002).

#### Aphid Individual Growth

These tests (**Figure 1B**) were performed with seedlings under the same controlled conditions as described above for plant propagation in the biotron. Two days after planting, a Perspex cylinder (5 cm long and 2 cm in diameter) was slipped over each seedling allowing the plant to grow through. Eight days after planting, five new-born nymphs were placed in the cylinder cage at the base of each plant and the top of the cage was sealed with cotton wool. The nymphs had been born on oats by winged females collected in the rearing cages the day before. After 4 d of growth, the nymphs were weighed individually on a Mettler M3 micro balance. Each plant genotype was replicated four times with one plant for each replicate.

#### Aphid Individual Growth After Pre-Infestation

This test (**Figure 1C**) was performed in the greenhouse chamber, under the same conditions as for aphid rearing described above. After planting, each pot was covered with a perforated plastic bag to hinder premature aphid infection. Eleven or 12 d after planting, a plastic cylinder cage (4.5 cm long and 2.5 cm diameter) was placed around the mid-section of the youngest fully developed leaf and kept in place by sponges with a slit for the leaf at the bottom and top of the cage. The cage was attached with a rubber band to a flower stick for support. Twenty aphids at different developmental stages were released in each cage in the pre-infestation treatment with the intention to try to accentuate callose induction. The cages in the non-infested treatment were left without aphids. After 3 d, all the aphids were removed with a soft brush and five newborn nymphs were released in each cage of both pre-infested and non-infested plants. From here on, the experiment was performed as in the aphid individual growth experiment. Each line and treatment was represented by six plants.

#### Aphid Population Growth

This test (**Figure 1D**) was performed in the greenhouse chamber under the conditions described above for aphid rearing. The experiment started with winged females released on oat seedlings in a 10 cm-diameter pot covered with a Perspex cylinder (19.5 cm tall and 6.5 cm in diameter). The day after, newborn nymphs were transferred to new oat seedlings in the same type of cylinder cage. Four days later, nymphs, now close to adulthood, were transferred singly to mutated and control barley plants, which had been grown for 12 or 13 d after planting. Each genotype was replicated 10 times with one plant and founder female for each replicate. The plants were covered with a perforated plastic bag (80 x 36 cm; Cryovac SM57OY, Baumann Saatzuchtbedarf GMBH, Germany) from planting and onwards, to avoid premature aphid infestation, and to keep the test aphid and its offspring on the test plant. The number of aphids per plant was counted 15 d after female release and the weight of the whole aphid population was recorded after drying at 30˚C for 3 d.

#### Aphid Host Choice

This test (**Figure 1E**) was performed in the greenhouse chamber under the same conditions as described above for aphid rearing. In a 10 cm-diameter pot, one control and one mutant seedling were planted, opposite to each other, 1 cm from the pot wall. Since aphids commonly move toward the light before locating a nearby plant to feed on, every second pot in a row was turned 180˚ in relation to the previous one. The pot was covered with a perforated plastic bag to hinder premature aphid infestation and to keep the test aphids confined. One week after planting, 10 apterous adults or nymphs close to adulthood were released on the soil in the middle of the pot. The test included four double mutant lines along with the control plants. Each mutant and control combination was represented by 16 pots, namely 16 replicates. One day later, the released aphids were counted, excluding nymphs that had been born on the plants.

#### Statistical Analyses

ANOVAs were performed using the software STATISTICA v. 9.1. For details of the ANOVAs see Tables, Figures and **Supplementary Table S1**.

## Results

### Sequences of the 1636 and 1639 Genes in GP

The 1636 gene has one intron while 1639 has no intron in GP, as previously shown for barley cv. Morex. The cDNA of 1636 shows 98.7% homology with the coding sequence of the glucanase gene with accession no. X67099 and 1639 shows 98.2% homology with the coding sequence of the glucanase gene with accession no. AK248899.

### Phylogenetic Relationships of Putative *β-1,3*-Glucanase Genes and Localization of the Glucanases in Barley

**Figure 2:**
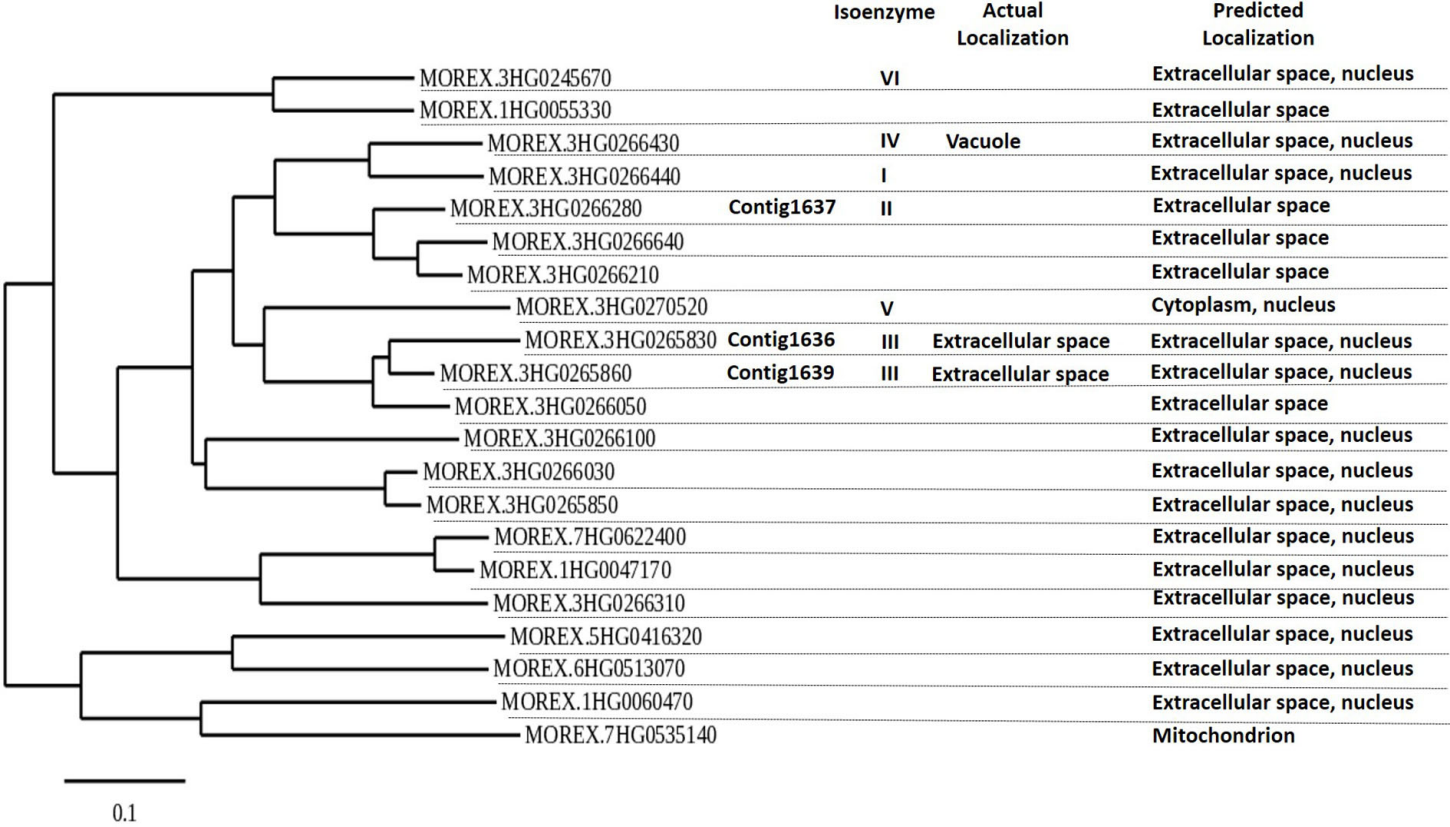
Phylogenetic tree of putative barley *β*-1,3-glucanase genes based on barley cultivar Morex DNA sequence, using Phylogeny.fr (maximum likelihood with bootstraps) software. *β*-1,3-Glucanase contigs 1636, 1637, and 1639 are indicated. *β*-1,3-Glucanase Morex sequences were obtained on December 2019 after blasting 1636, 1637, and 1639 in IPK’s barley blast server (http://webblast.ipk-gatersleben.de/barley_ibsc/). Actual isoenzyme information and subcellular protein localization refer to information by Li et al. (1996) and UniProt.org. Predicted localization of expression is from Bologna unified subcellular component annotator (BUSCA) (http://busca.biocomp.unibo.it).

The phylogenetic analysis of all putative *β-1,3*-glucanase genes in the genome sequence of Morex showed 21 putative *β-1,3*-glucanase genes, clustering in three main groups. The genes 1636, 1639, and 1637 all belong to the same main gene clade, but the 1636 and 1639 sequences are most similar (**Figure 2**). All three are expected to produce proteins that are excreted extracellularly, like most of the other *β-1,3*-glucanases (**Figure 2**; Li et al., 1996; Roulin et al., 1997).

### Gene Expression Levels With and Without Aphid Infestation

**Figure 3:**
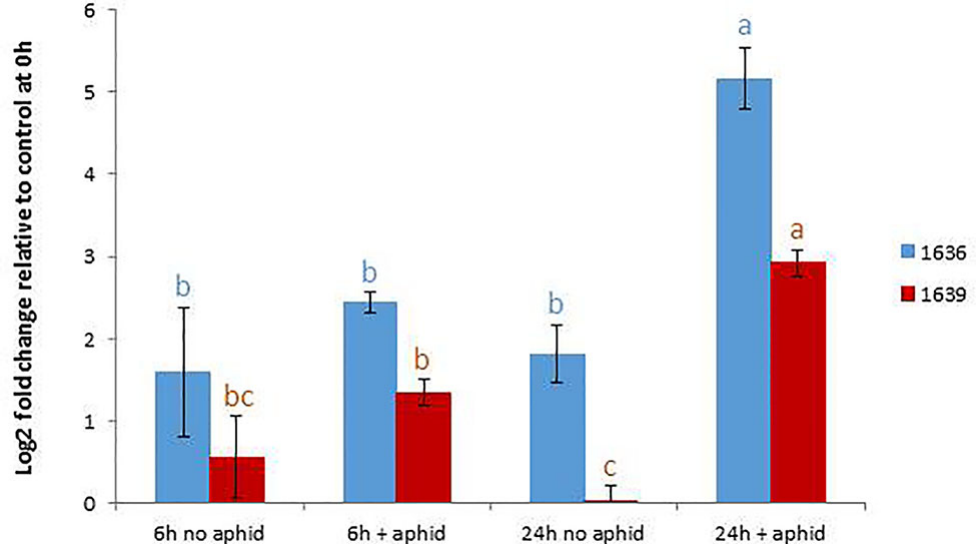
Relative gene expression levels (mean ± SE, n=5) of 1636 and 1639 in Golden Promise, expressed as fold change relative to their expressions at the start of the experiment and normalized against the changes in expression level of the reference genes SF427 and Hsp70. The data were analyzed by one-way ANOVA (see **Table S1**), followed by Tukey’s multiple comparison test for each gene separately. Different letters above bars indicate significant differences at *p*=0.05.

**Figure 4:**
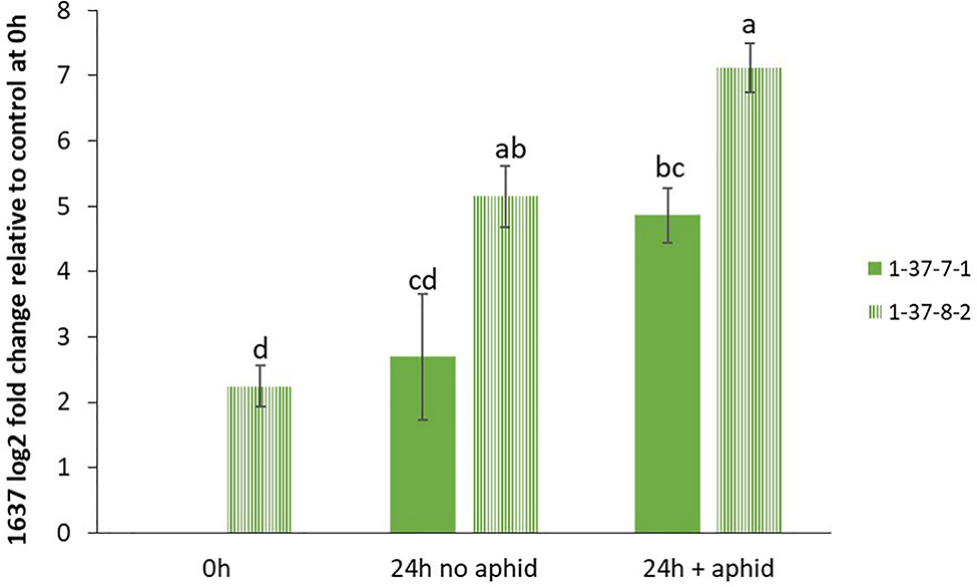
Relative gene expression levels (mean ± SE, n=4) of *β*-1,3-glucanase gene 1637, expressed as fold change relative to its expression in the control line 1-37-7-1 at the start of the experiment and normalized against changes in expression level of the reference genes SF427 and Hsp70. The data were analyzed by one-way ANOVA (see **Table S1**), followed by Tukey’s multiple comparison test. Different letters above bars indicate significant differences between the double mutant line 1-37-8-2 and control line 1-37-7-1 or between different treatments at *p*=0.05.

In order to confirm the expression levels of the target genes in GP to be used for mutagenesis, we performed qRT-PCR analyses of the genes 1636 and 1639 with or without aphid infestation. Both transcripts showed significant upregulation upon aphid infestation for 24 h (**Figure 3**). In order to test if the 1637 gene expression was different in a mutant homozygous for 1636 and 1639 mutations compared to a non-mutated genotype, the induction experiment was also carried out with a double mutant in comparison with a control line (see line production below). The mutant line had a significantly higher expression of 1637 than the control line, without as well as with the aphid treatment. There was a tendency of, but not a significant upregulation of 1637 by *R. padi* in both genotypes (**Figure 4**). We interpret the increase of all three transcripts with time in the treatments without aphids to be a plant reaction to the cages applied to the leaves.

### Molecular Characteristics of Mutants

**Figure 5:**
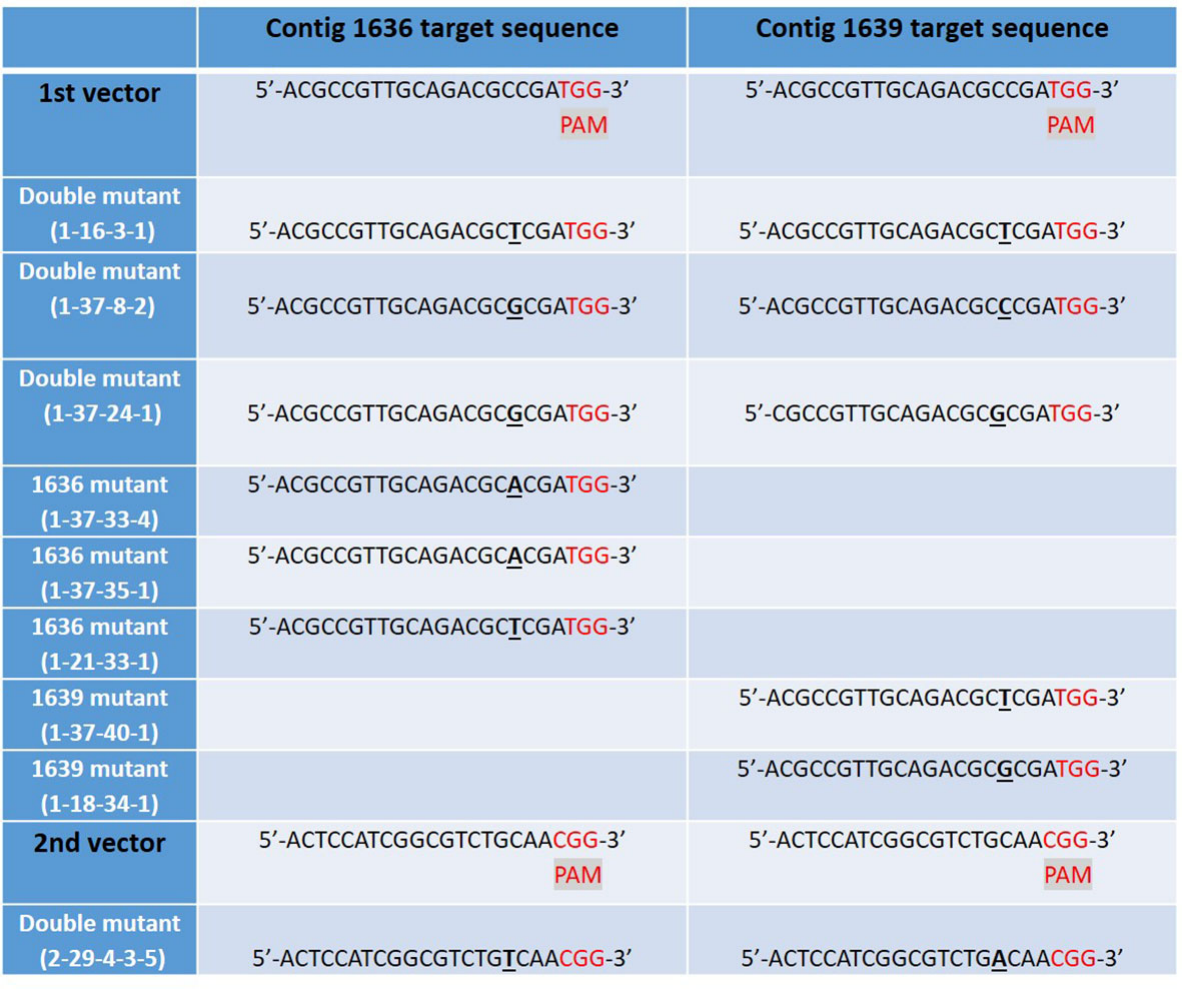
The mutated CRISPR/Cas9 target sites of *β*-1,3-glucanase gene sequences in nine studied lines of barley cv Golden Promise, derived from five different transgenic plants: 1-16, 1-37, 1-21, 1-18, and 2-29. Single DNA base inserts are shown in underlined bold text and a protospacer adjacent motif (PAM) site in red. Two different vectors were used to generate the mutant lines, each targeting the two *β*-1,3-glucanase genes 1636 and 1639 resulting in lines with one or both target genes mutated.

We obtained 76 plantlets (T_0_) from JIC, which had been confirmed transgenic by PCR analysis for the hygromycin resistance gene. Mutations in the transgenic lines of GP were first screened by HRFA whereby DNA base insertions or deletions in the target regions could be identified, down to one base pair (bp) indel (Andersson et al., 2017). Twenty one of the 76 T_0_ plants showed mutations in the target genes, of which, 20 showed mutation in one of the two target genes, while one showed mutations in both genes. Offspring produced by selfing (S) of several mutant T_0_ lines were further screened for mutations in the target genes by HRFA, followed by sequencing in the subsequent generations to bring plants to homozygosity. Meanwhile, the segregation also resulted in lines that do not have any mutations in the target genes, and such lines were used as controls in the study. All confirmed mutant lines used in subsequent plant characterizations and aphid tests had 1 bp insertion, which had occurred only in the target sequences common to both genes and for both vectors (**Figure 5**). Furthermore, all lines used in the aphid tests were CRISPR/Cas9-vector free as determined by PCR analysis for the hygromycin resistance gene in the transformation vectors.

Hereafter, we refer to lines as single 1636, single 1639, or double mutants. Designations for lines are as follows: the first digit represents transformation vector 1 or 2. The second digit denotes the original transgenic plant no. (tissue-cultured plant T_0_). The third digit denotes the no. of an S_1_ plant obtained from selfing of T_0_. S_1_ plants were further selfed, for one (fourth digit) or two (fifth digit) generations to obtain homozygous lines for further analyses.

**Figure 6:**
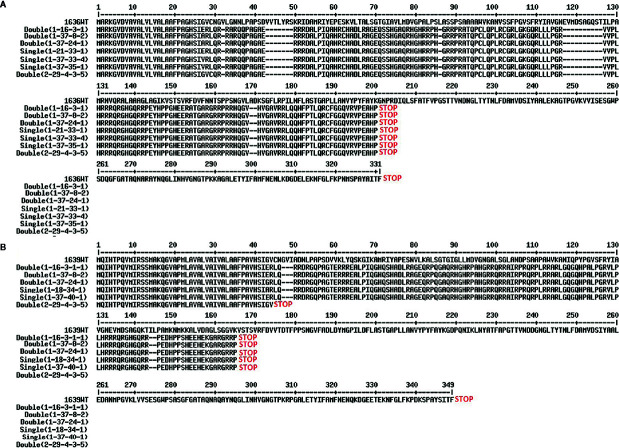
Predicted proteins from the 1636 **(A)** and 1639 **(B)** mutant lines. Translation of genes from cultivar Morex wild type (WT) is compared with translations of the nine mutant lines.

Four double mutant (1-16-3-1, 1-37-8-2, 1-37-24-1, and 2-29-4-3-5), three 1636 single mutant (1-37-33-4, 1-37-35-1 and 1-21-33-1), and two 1639 single mutant (1-37-40-1 and 1-18-34-1) lines were characterized further. All these mutants had a single base insertion (**Figure 5**) resulting in a frame shift causing a premature stop codon that might lead to a non-functional protein. All but one of the mutants had a stop codon at the same site (**Figure 6**). The 2-29-4-3-5 double mutant had it much earlier in the DNA sequence of the 1639 gene, expected to result in a protein 121 amino acids shorter than the others’ (**Figure 6**).

### *β*-1,3-Glucanase Enzymatic Activity in Mutant Lines

**Figure 7:**
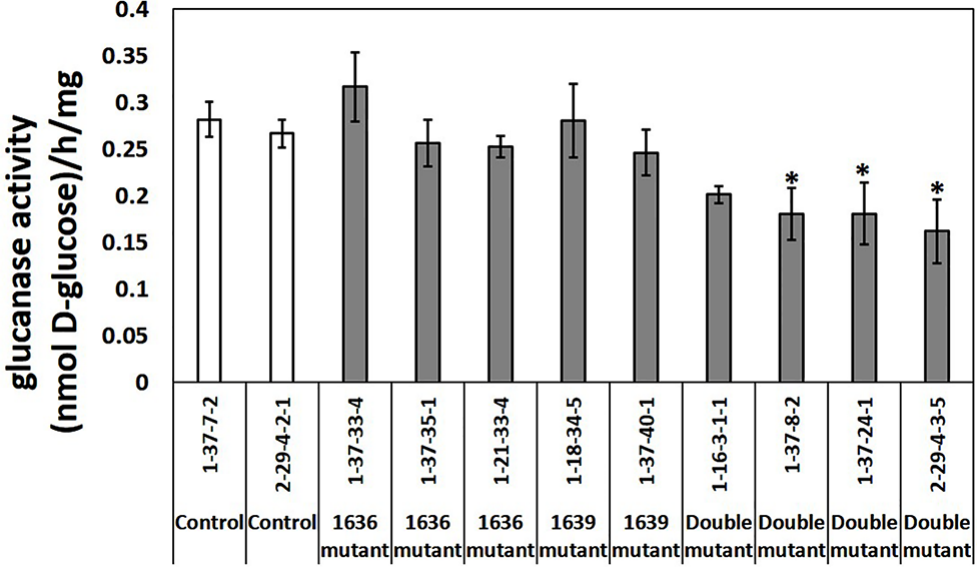
Total *β*-1,3-glucanase activities (mean ± SE, n=4 or 5) in crude protein extracts of seedlings from two control lines, five single and four double mutant barley lines. The data was analyzed by one-way ANOVA (see **Table S1**), followed by LSD test at *p*=0.05. * above the bar indicate significant differences between the mutant line and both control lines.

Total *β*-1,3-glucanase enzymatic activity was measured in an assay using laminarin as substrate. Three out of four double mutant lines had significantly lower *β-1,3*-glucanase activity than the two controls, up to 40% lower, whereas the single mutants did not differ in enzymatic activity compared to that of the controls (**Figure 7**).

### Callose in Mutant Lines

**Figure 8:**
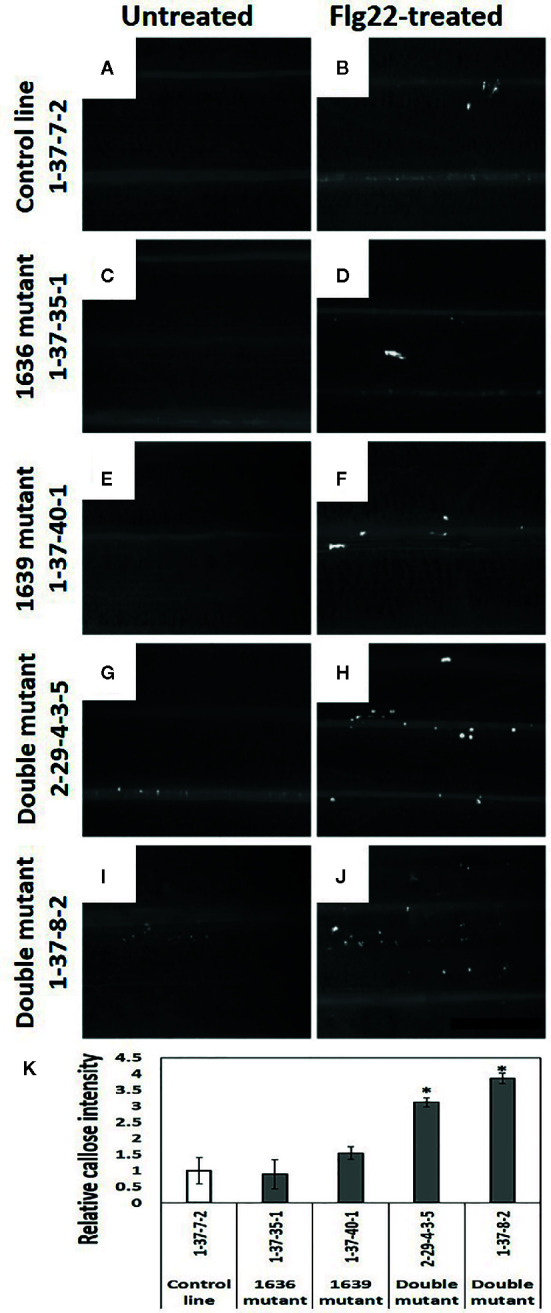
Callose deposition (white spots in the images) in barley leaves of 2 weeks old seedlings, without **(A, C, E, G, I)** and with Flg22 treatment **(B, D, F, H, J)**. Callose was stained with aniline blue. a and b=control line 1-37-7-2, c and d=1636 mutant line 1-37-35-1, e and f=1639 mutant line 1-37-40-1, g and h=double mutant line 2-29-4-3-5, and i and j=double mutant line 1-37-8-2. Scale bar 500 μm. **(K)** Number of pixels corresponding to the color of callose in Flg22-treated leaves of mutant lines relative to that of a control line (mean ± SE, n=4). The data was analyzed by one-way ANOVA (see **Table S1**), followed by LSD test at *p*=0.05. * above the bar indicate significant differences between the mutant and the control line.

Leaves of double mutant plants treated with flagellin had significantly more callose than the plants of 1636 or 1639 single mutant or control lines (**Figure 8K**). Also non-treated plants of double mutants tended to have more callose than plants of the other two categories (**Figures 8A–J**).

Double and single mutants did not show any morphological differences compared to control lines.

### Aphid Responses

#### Aphid Individual Growth

Single or double mutant lines from five different transformation events representing both vectors were tested for how well they supported aphid growth, experiments with lines from T_0_ 1–18 and 1–16 replicated over generations and lines from T_0_ 2–29 replicated three times. Reduced growth is an indicator of plant resistance. No significant differences were found between the control and mutant lines with regards to individual weight of aphids after 4 d of nymphal growth on the test plants (**Table 2**).

**Table 2:** *Rhopalosiphum padi* individual weight (mean ± SE, n=4) after 4 d of growth on test plants.

Plant type	Line no. (generation S_2_)	Aphid weight (µg)	*p-value*	Plant type	Line no. (generation S_3_ or S_4_)	Aphid weight (µg)	*p-value*
				Control ^a^	1-37-7-1	270 ± 19	*0.19*
				Control	1-37-7-2	315 ± 28	
				Double mutant	1-37-8-2	329 ± 31	
				Double mutant	1-37-24-1	260 ± 27	
				Mutant 1636	1-37-33-4	330 ± 46	
Mutant 1636	1-37-35					250 ± 21	
				Mutant 1639	1-37-40-1	293 ± 33	
				Control	2-29-4-2-1	302 ± 24	*0.14*
				Double mutant	2-29-4-3-5	251 ± 13	
				Control	2-29-4-2-1	363 ± 45	*0.18*
				Double mutant	2-29-4-3-5	307 ± 40	
				Control ^b^	2-29-4-2-1	263 ± 18	*0.28*
				Double ^b^ mutant	2-29-4-3-5	302 ± 18	
Control	1-18-46	414 ± 78	*0.14*	Control	1-18-46-1	264 ± 39	*0.29*
Mutant 1639	1-18-34	508 ± 35		Control	1-18-46-2	341 ± 68	
				Mutant 1639	1-18-34-1	251 ± 44	
				Mutant 1639	1-18-34-3	257 ± 16	
Control	1-21-31	541 ± 48	*0.31*				
Mutant 1636	1-21-33	463 ± 35					
Control	Mean^c^	436 ± 40	*0.08*	Control	Mean^c^	298 ± 22	*0.29*
Double mutant	1-16-3	402 ± 36		Double mutant	1-16-3-1	236 ± 28	
				Double mutant	1-16-3-2	249 ± 46	

Each plant (replicate) was infested with five newborn nymphs. Differences between control and mutant lines were tested for by ANOVA (see **Table S1**) based on replicate means of individual aphid weights.

^a^Control lines originated from the same T_0_ from which mutation lines were segregated, except for T_0_ 1–16 where no such control line was obtained.

^b^Aphids of Alnarp origin. All the other tests were made with aphids originating from Saxtorp.

^c^Means for other controls (from T_0_ origins 1–18 and 1–21 or 1–18 and 1–37 tested simultaneously) were calculated per replicate and used in statistics for the mutants from T_0_ 1–16.

#### Aphid Individual Growth After Pre-Infestation

Single 1636, single 1639, and double mutant lines from the T_0_ no. 1–37 were compared with control lines that had segregated out as non-mutated. There was no significant difference in individual nymphal weight between plants with and without aphid infestation prior to the 4-d long aphid growth test (**Table 3**). Moreover, there was no significant difference in individual aphid weight between mutant and control lines in this test either.

**Table 3:** *Rhopalosiphum padi* individual weight (mean ± SE, n=6) after 4 d of growth on the test plants that had been either pre-infested with 20 aphids (all aphids removed after 3 d) or without pre-infestation.

Plant type^a^	Line no.	Aphid weight (µg)	Aphid weight (µg)
		Pre-infested	Not pre-infested
Control^b^	1-37-7-1	239 ± 17	241 ± 17
Control	1-37-7-2	215 ± 14	219 ± 11
Double mutant	1-37-8-2	237 ± 19	226 ± 18
Double mutant	1-37-24-1	223 ± 7	266 ± 12
Mutant 1636	1-37-33-4	227 ± 24	210 ± 11
Mutant 1639	1-37-40-1	239 ± 20	236 ± 20

Each plant (replicate) was infested with five newborn nymphs. There were no significant aphid weight differences among the lines (p=0.36) or between the pre-infestation treatments (p=0.75) when tested by ANOVA (see **Table S1**) based on replicate means of individual aphid weights.

^a^Lines were tested in generation S_3._

^b^Control lines originated from the same T_0_ plant 1–37 from which mutations were segregated.

#### Aphid Population Growth

The same lines that were tested for individual aphid growth after pre-infestation were also tested for aphid population growth after release of one founder female per test plant. There was no significant difference in aphid population size or aphid biomass weight between any of the lines after 15 d of aphid reproduction (**Table 4**).

**Table 4:** Aphid population size (mean ± SE) 15 d after release of one female *R. padi* per plant.

Plant type^a^	Line no.	No. replicates	No. of aphids	*p-value*	Dry weight of aphid colony (mg)	*p-value*
Control^b^	1-37-7-1	7	299 ± 44	*0.11*	32.2 ± 4.6	*0.07*
Control	1-37-7-2	10	275 ± 47		28.3 ± 5.5	
Double mutant	1-37-8-2	10	393 ± 35		42.1 ± 3.1	
Double mutant	1-37-24-1	9	271 ± 48		26.1 ± 5.5	
Mutant 1636	1-37-33-4	8	404 ± 44		42.0 ± 4.5	
Mutant 1639	1-37-40-1	9	307 ± 61		29.5 ± 5.7	

There were initially 10 plants (replicates), but this number was reduced due to death of the founder female in four of the six lines. There were no significant differences in mean number of aphids or mean aphid biomass when tested by ANOVA (see **Table S1**).

^a^Lines were tested in generation S_3._

^b^Control lines originated from the same T_0_ plant from which mutations were segregated.

#### Aphid Host Choice

Pairs of control and double mutant lines from three different T_0_ were compared with regards to aphid host choice one day after infestation. Reduced aphid acceptance of a plant is another type of indicator for plant resistance. In one of the comparisons aphids were significantly more abundant on the mutant than on the control line but when retested there was no significant preference (**Table 5**).

**Table 5:** Number of aphids (Mean ± SE, n=16) on a mutant and a control plant grown in the same pot.

No. of aphids	Double mutant^b^	Paired t-test
Control^ab^		*t-value p-value*
1-37-7-1	1-37-8-2	
2.1 ± 0.4 3.4 ± 0.4	4.0 ± 0.4 3.6 ± 0.4	*3.00 0.01* *0.20 0.84*
1-37-7-1	1-37-24-1	
2.8 ± 0.4	3.4 ± 0.5	*0.84 0.41*
1-37-7-1	1-16-1-1-1	
2.8 ± 0.3	2.2 ± 0.3	*1.25 0.23*
2-29-4-2-1	2-29-4-3-5	
2.8 ± 0.4	2.5 ± 0.4	*0.39 0.71*

Counts were made 24 h after release of 10 apterous adults, or nymphs close to adulthood.

^a^Control lines originated from the same T_0_ plant from which mutations were segregated, except for test of mutant line 1-16-1-1-1.

^b^Lines were tested in generations S_3_ or S_4_.

## Discussion

In the present study there was no support for our expectation that performance of *R. padi* would be hampered by mutations in two of the three glucanase genes previously found to be upregulated by this aphid in various barley germplasm (Delp et al., 2009; Saheed et al., 2009; Mehrabi et al., 2016), and here also found upregulated in the GP cultivar. This was first tested in the populations derived from five different CRISPR/Cas9 transformation events of GP representing four different single base insertions in 1636 and 1639, respectively, using a standard test for aphid individual growth. This particular test method has previously been used for resistance screening in a barley breeding program and has been confirmed to successfully select for *R. padi* resistance, as determined by reduced population growth in a field test as well as by molecular markers for the resistance (Åhman and Bengtsson, 2019). However, since this test lasted only for 4 d and included just five young aphids per cage and plant, we repeated it but with addition of a pre-infestation treatment of the plants with 20 aphids that were removed after 3 d when the actual test was started with newborn nymphs. The rationale for this was that the high aphid density at pre-infestation was expected to induce so much callose in the phloem vessels that the plants mutated in one or two *β-1,3*-glucanase genes would not be able to degrade it to the extent that normal plants do, which in turn would reduce phloem sap access and aphid growth. However, this pre-infestation did not affect the result as no significant effect on aphid growth was found when comparing the single or double mutant lines with the controls from the same family. The same lines were also subjected to an *R. padi* population growth test, which lasted for 15 d, but again no significant differences between mutant and control lines were found. Finally, we performed a choice test to investigate if *R. padi* was able to detect the effects of these mutations. Here again no significant difference in aphid preference was found between the mutant and control lines except for one combination, which showed that the aphids preferred the mutant. However, since this result could not be reproduced, it might be a statistical Type I error.

Plant analyses showed that total *β*-1,3-glucanase enzymatic activity was significantly reduced in three of the four double mutant lines tested. Presence of callose in leaves tended to be more predominant constitutively in the double mutant lines compared to single mutant and control lines. This was further accentuated when the plants were provoked with the Flg22 peptide from bacterial flagellin, a protein known to trigger callose deposition (Gomez-Gomez et al., 1999). However, as described above, this increased callose deposition in the double mutant lines did not affect the *R. padi* performance and host preference.

There are many *β*-1,3-glucanase genes in barley. Saheed et al. (2009) identified 16 unigenes for *β-1,3*-glucanases for barley in the NCBI database and Li et al. (1996) showed seven genes for *β*-1,3-glucanase isoenzymes to be located on the long arm of chromosome 3H. In our study, through blasting the DNA sequences of 1636, 1637, and 1639 against the genome sequence of cv. Morex, we distinguished 21 putative *β*-1,3-glucanase genes, some of which clustering closely with the three genes. 1636 and 1639 align with genes coding for proteins belonging to the isoenzyme group GIII and 1637 with a gene coding for a protein in isoenzyme group GII. GII and GIII isoenzymes are basic proteins, which are secreted extracellularly (Li et al., 1996; Roulin et al., 1997). Various barley pathogens such as *Blumeria graminis* (Jutidamrongphan et al.,1991; Ignatius et al., 1994), *Bipolaris sorokiniana* (Jutidamrongphan et al., 1991), and *Rhynchosporium secalis* (Roulin et al., 1997) have been shown to upregulate GII and GIII isoenzyme genes and the promoter of GIII isoenzyme genes is activated by the plant defense hormone salicylic acid (Li et al., 2005).

The reason why we concentrated on the three *β*-1,3-glucanase genes 1636, 1639, and 1637 was that they have been shown to be upregulated by *R. padi* in previous studies even if the expression levels of the three genes differed depending on barley genotype and the methods used for analyses. In a microarray study, Delp et al. (2009) found 1636 to be more upregulated in two susceptible than in two partially resistant cultivars, while 1637 was upregulated in all four. The gene 1639 and another gene were expressed constitutively in the susceptible lines and two other genes were expressed constitutively in the resistant lines. Mehrabi et al. (2016) found 1639 and 1637 to be more upregulated in the susceptible breeding lines in all comparisons where there were significant differences between susceptible and resistant lines. In total, 15 lines were analyzed and all of them expressed genes 1639 and 1637 and aphids significantly upregulated 1639 in 13 of the lines and 1637 in seven of the lines. The gene 1636 was expressed in only five of the 14 successfully analyzed lines with upregulation in four lines (Mehrabi et al., 2016). Saheed et al. (2009) analyzed 12 out of 16 glucanase genes in the aphid-susceptible cv. Clipper and found 1636 and 1637 to be upregulated by *R. padi* and 1639 constitutively expressed along with two other genes (whereas all five were strongly upregulated by the aphid *D. noxia*). From this spectrum of barley glucanase gene responses to *R. padi* there is reason to believe that the glucanase coded for by the gene 1637 might also play a role in aphid resistance. In GP with mutations both in 1636 and 1639, 1637 was higher expressed than in the control line, something which might potentially compensate for reduced expressions of 1636 and 1639.

At present it is unknown if there are certain *β*-1,3-glucanases in barley that are predominantly localized to sieve elements, a localization which would be most relevant for influencing aphid performance. Since sieve element cells lack nuclei, glucanases in phloem must be produced in adjacent cells (Van Bel and Will, 2016). Glucanases from all three genes studied here are known to be localized extracellularly, but one might speculate as to whether 1637, and not 1636 and 1639, has such phloem specificity and if this might explain the absence of effects from the double mutants on *R. padi* resistance. Regarding tissue specificity of callose synthases, Glucan Synthase-Like7 (GSL7) in Arabidopsis has been shown to be responsible for callose deposition in sieve pores (Barratt et al., 2011; Xie et al., 2011).

The extent of callose deposition as a plant response to aphid infestation differs depending on aphid and plant species, as well as aphid and plant genotype. Aphid/plant combinations where callose deposition is more extensive are *D. noxia* infesting barley (Saheed et al., 2009) and wheat (Van der Westhuizen et al., 1998; Van der Westhuizen et al., 2002), *M. persicae* infesting resistant *Capsicum baccatum* (Sun et al., 2018b), and *Macrosiphum euphorbiae* Thomas in potato, although in this latter case callose accumulation was less in the apoplast and phloem sieve tubes where the aphids resided than in distal plant parts (Samaha, 2017). This is in line with our hypothesis, that aphids reduce callose locally, potentially *via* induced upregulation of plant *β*-1,3-glucanases. An Arabidopsis mutant study suggests that callose deposition is a resistance factor and that especially one of the *β*-1,3-glucanases studied play a role in plant susceptibility to *M. persicae* (Shoala et al., 2018). There are also examples of interactions between secondary metabolites and callose build-up. Activation of the defense compounds DIMBOA-glucoside in maize (Ahmad et al., 2011) and a methylated indolyl glucosinolate in Arabidopsis (Clay et al., 2009) triggers callose deposition which in the maize case correlates with resistance to the aphid *Rhopalosiphum maidis* (Fitch) (Meihls et al., 2013). *R. padi* infestation causes less callose deposition than *D. noxia* on the same susceptible barley host, but in none of the cases was this found to be due to regulation of the callose synthesis genes by the aphids. Neither was it a result of stronger *β*-1,3-glucanase gene upregulation by *R. padi* than by *D. noxia*, but rather to the difference in callose synthase activation by the two aphids (Saheed et al., 2009). Will and Vilcinskas (2015) suggest that the predominant protein in aphid gelling saliva and the resulting hardened stylet sheet prevent sieve-tube occlusion which would otherwise result from calcium influx from the apoplast into the aphid-damaged sieve element. However, stylet sheet formation is typical for all aphids, including *D. noxia.* This suggests that the callose-inducing signal in the *D. noxia* interaction with barley is phloem-transported (Saheed et al., 2009). The localization of *β*-1,3-glucanases as a result of *D. noxia* infestation was concentrated to cell walls of the vascular bundles but more so in resistant than in susceptible plants (Van der Westhuizen et al., 2002).

Callose deposition in plants is induced by various stress factors such as mechanical wounding, high temperature, certain chemicals and infestation by plant pathogens, including viruses. Similar to our hypothesis, pathogen induction of plant *β-*1,3-glucanases might counteract this callose defense. However, Zavaliev et al. (2013) found that only the constitutively expressed *β-*1,3-glucanase localized to plasmodesmata, and not the virus-induced type, influenced tobamovirus cell to cell movement in Arabidopsis.

Through mutations in two of the three *β*-1,3-glucanase genes, commonly found to be upregulated by *R. padi* in barley susceptible to this aphid, we were unable to confirm the hypothesis that these proteins function as major aphid susceptibility factors. However, to completely reject this hypothesis it is necessary to also study barley mutants in which the third *β*-1,3-glucanase gene has been knocked out. Moreover, deeper knowledge about the cellular localization of the *β*-1,3-glucanase gene expressions and protein depositions would help further interpretation of our results.

## Data Availability Statement

The raw data supporting the conclusions of this article will be made available by the authors, without undue reservation, to any qualified researcher.

## Author Contributions

IÅ suggested the hypothesis and led the project together with L-HZ. S-YK propagated, analyzed and described the plant material molecularly and biochemically, with the exception of RT-qPCR analyses of the target genes performed by TB and HRFA analyses performed by NO. IÅ planned and VH performed the aphid tests. IÅ and S-YK wrote the main part of the manuscript. NO and TB wrote their parts and L-HZ contributed improvements to the whole manuscript.

## Conflict of Interest

The authors declare that the research was conducted in the absence of any commercial or financial relationships that could be construed as a potential conflict of interest.
