# Supplementary material for: Mutations in Two Aphid-Regulated β-1,3-Glucanase Genes by CRISPR/Cas9 Do Not Increase Barley Resistance to Rhopalosiphum padi L
Source: Front Plant Sci. 2020 Jul 9;11:1043. doi: 10.3389/fpls.2020.01043 (PMC7381296; doi:10.3389/fpls.2020.01043)
Supplement: Supplementary file 1 [file DataSheet_1.docx]

Table S1. ANOVA results for data in Tables 2, 3 and 4, and Figures 3, 4, 7 and 8, using software STATISTICA v. 9.1

**Table 2:**

| Univariate Tests of Significance for Individual Aphid Weight (**1-37**) Sigma-restricted parameterization Effective hypothesis decomposition | | | | | |
| --- | --- | --- | --- | --- | --- |
|  | **SS** | **Degr. of - Freedom** | **MS** | **F** | **p** |
| **Intercept** | 2,392066 | 1 | 2,392066 | 904,7570 | 0,000000 |
| **Line** | 0,026258 | 6 | 0,004376 | 1,6553 | 0,189562 |
| **Replicate** | 0,030328 | 3 | 0,010109 | 3,8236 | 0,027921 |
| **Error** | 0,047590 | 18 | 0,002644 |  |  |

| Univariate Tests of Significance for Individual Aphid Weight (**2-29-4-3-5**) Sigma-restricted parameterization Effective hypothesis decomposition | | | | | |
| --- | --- | --- | --- | --- | --- |
|  | **SS** | **Degr. of - Freedom** | **MS** | **F** | **p** |
| **Intercept** | 0,613278 | 1 | 0,613278 | 464,4434 | 0,000219 |
| **Line** | 0,005151 | 1 | 0,005151 | 3,9010 | 0,142737 |
| **Replicate** | 0,004768 | 3 | 0,001589 | 1,2037 | 0,441233 |
| **Error** | 0,003961 | 3 | 0,001320 |  |  |

| Univariate Tests of Significance for Individual Aphid Weight (**2-29-4-3-5**) Sigma-restricted parameterization Effective hypothesis decomposition | | | | | |
| --- | --- | --- | --- | --- | --- |
|  | **SS** | **Degr. of - Freedom** | **MS** | **F** | **p** |
| **Intercept** | 0,897130 | 1 | 0,897130 | 432,0482 | 0,000244 |
| **Line** | 0,006328 | 1 | 0,006328 | 3,0476 | 0,179197 |
| **Replicate** | 0,037163 | 3 | 0,012388 | 5,9658 | 0,088255 |
| **Error** | 0,006229 | 3 | 0,002076 |  |  |

| Univariate Tests of Significance for Individual Aphid Weight (**2-29-4-3-5**) Sigma-restricted parameterization Effective hypothesis decomposition | | | | | |
| --- | --- | --- | --- | --- | --- |
|  | **SS** | **Degr. of - Freedom** | **MS** | **F** | **p** |
| **Intercept** | 0,637885 | 1 | 0,637885 | 365,7336 | 0,000312 |
| **Line** | 0,003003 | 1 | 0,003003 | 1,7219 | 0,280825 |
| **Replicate** | 0,002246 | 3 | 0,000749 | 0,4293 | 0,747255 |
| **Error** | 0,005232 | 3 | 0,001744 |  |  |

| Univariate Tests of Significance for Individual Aphid Weight (**1-18**) Sigma-restricted parameterization Effective hypothesis decomposition | | | | | |
| --- | --- | --- | --- | --- | --- |
|  | **SS** | **Degr. of - Freedom** | **MS** | **F** | **p** |
| **Intercept** | 1,699246 | 1 | 1,699246 | 397,5969 | 0,000276 |
| **Line** | 0,017578 | 1 | 0,017578 | 4,1130 | 0,135598 |
| **Replicate** | 0,074011 | 3 | 0,024670 | 5,7725 | 0,091936 |
| **Error** | 0,012821 | 3 | 0,004274 |  |  |

| Univariate Tests of Significance for Individual Aphid Weight (**1-18**) Sigma-restricted parameterization Effective hypothesis decomposition | | | | | |
| --- | --- | --- | --- | --- | --- |
|  | **SS** | **Degr. of - Freedom** | **MS** | **F** | **p** |
| **Intercept** | 1,237656 | 1 | 1,237656 | 258,5318 | 0,000000 |
| **Line** | 0,021092 | 3 | 0,007031 | 1,4686 | 0,287372 |
| **Replicate** | 0,057534 | 3 | 0,019178 | 4,0061 | 0,045816 |
| **Error** | 0,043085 | 9 | 0,004787 |  |  |

| Univariate Tests of Significance for Individual Aphid Weight (**1-21**) Sigma-restricted parameterization Effective hypothesis decomposition | | | | | |
| --- | --- | --- | --- | --- | --- |
|  | **SS** | **Degr. of - Freedom** | **MS** | **F** | **p** |
| **Intercept** | 2,014025 | 1 | 2,014025 | 247,2359 | 0,000559 |
| **Line** | 0,012325 | 1 | 0,012325 | 1,5129 | 0,306353 |
| **Replicate** | 0,017536 | 3 | 0,005845 | 0,7176 | 0,604207 |
| **Error** | 0,024438 | 3 | 0,008146 |  |  |

| Univariate Tests of Significance for Individual Aphid Weight (**1-16**) Sigma-restricted parameterization Effective hypothesis decomposition | | | | | |
| --- | --- | --- | --- | --- | --- |
|  | **SS** | **Degr. of - Freedom** | **MS** | **F** | **p** |
| **Intercept** | 1,014348 | 1 | 1,014348 | 2855,978 | 0,000350 |
| **Line** | 0,003700 | 1 | 0,003700 | 10,418 | 0,084061 |
| **Replicate** | 0,026104 | 3 | 0,008701 | 24,500 | 0,039471 |
| **Error** | 0,000710 | 2 | 0,000355 |  |  |

| Univariate Tests of Significance for Individual Aphid Weight (**1-16**) Sigma-restricted parameterization Effective hypothesis decomposition | | | | | |
| --- | --- | --- | --- | --- | --- |
|  | **SS** | **Degr. of - Freedom** | **MS** | **F** | **p** |
| **Intercept** | 0,817452 | 1 | 0,817452 | 288,4531 | 0,000003 |
| **Line** | 0,008579 | 2 | 0,004289 | 1,5135 | 0,293637 |
| **Replicate** | 0,023570 | 3 | 0,007857 | 2,7724 | 0,133097 |
| **Error** | 0,017003 | 6 | 0,002834 |  |  |

**Table 3.**

| Univariate Tests of Significance for Individual Aphid Weight (**1-37**) Sigma-restricted parameterization Effective hypothesis decomposition | | | | | |
| --- | --- | --- | --- | --- | --- |
|  | **SS** | **Degr. of - Freedom** | **MS** | **F** | **p** |
| **Intercept** | 3,858179 | 1 | 3,858179 | 2710,823 | 0,000000 |
| **Line** | 0,007995 | 5 | 0,001599 | 1,123 | 0,357770 |
| **Replicate** | 0,020027 | 5 | 0,004005 | 2,814 | 0,023868 |
| **Pre-infestion** | 0,000142 | 1 | 0,000142 | 0,100 | 0,753469 |
| **Error** | 0,085395 | 60 | 0,001423 |  |  |

**Table 4.**

| Univariate Tests of Significance for No. aphids (**1-37**) Sigma-restricted parameterization Effective hypothesis decomposition | | | | | |
| --- | --- | --- | --- | --- | --- |
|  | **SS** | **Degr. of - Freedom** | **MS** | **F** | **p** |
| **Intercept** | 5576320 | 1 | 5576320 | 306,6512 | 0,000000 |
| **Line** | 178897 | 5 | 35779 | 1,9676 | 0,105823 |
| **Replicate** | 241567 | 9 | 26841 | 1,4760 | 0,192094 |
| **Error** | 691014 | 38 | 18185 |  |  |

| Univariate Tests of Significance for Pop. dry weight (**1-37**) Sigma-restricted parameterization Effective hypothesis decomposition | | | | | |
| --- | --- | --- | --- | --- | --- |
|  | **SS** | **Degr. of - Freedom** | **MS** | **F** | **p** |
| **Intercept** | 58167,45 | 1 | 58167,45 | 267,3066 | 0,000000 |
| **Line** | 2445,28 | 5 | 489,06 | 2,2474 | 0,069266 |
| **Replicate** | 1976,30 | 9 | 219,59 | 1,0091 | 0,449952 |
| **Error** | 8269,02 | 38 | 217,61 |  |  |

**Figure 3.**

| Univariate Tests of Significance for Fold change (**1636**) Sigma-restricted parameterization Effective hypothesis decomposition | | | | | |
| --- | --- | --- | --- | --- | --- |
|  | **SS** | **Degr. of - Freedom** | **MS** | **F** | **p** |
| **Intercept** | 151,3851 | 1 | 151,3851 | 136,1043 | 0,000000 |
| **Treatment & time** | 40,8596 | 3 | 13,6199 | 12,2451 | 0,000205 |
| **Error** | 17,7964 | 16 | 1,1123 |  |  |

| Univariate Tests of Significance for Fold change (**1639**) Sigma-restricted parameterization Effective hypothesis decomposition | | | | | |
| --- | --- | --- | --- | --- | --- |
|  | **SS** | **Degr. of - Freedom** | **MS** | **F** | **p** |
| **Intercept** | 29,28681 | 1 | 29,28681 | 68,16231 | 0,000000 |
| **Treatment & time** | 23,99738 | 3 | 7,99913 | 18,61722 | 0,000018 |
| **Error** | 6,87461 | 16 | 0,42966 |  |  |

**Figure 4.**

| Univariate Tests of Significance for Fold change (**1637**) Sigma-restricted parameterization Effective hypothesis decomposition | | | | | |
| --- | --- | --- | --- | --- | --- |
|  | **SS** | **Degr. of - Freedom** | **MS** | **F** | **p** |
| **Intercept** | 365,4141 | 1 | 365,4141 | 387,7237 | 0,000000 |
| **Treatment & time & line** | 59,7523 | 4 | 14,9381 | 15,8501 | 0,000043 |
| **Error** | 13,1944 | 14 | 0,9425 |  |  |

**Figure 7.**

| Univariate Tests of Significance for Glucanase activity (**Control, single mutant, double mutant lines**) Sigma-restricted parameterization Effective hypothesis decomposition | | | | | |
| --- | --- | --- | --- | --- | --- |
|  | **SS** | **Degr. of - Freedom** | **MS** | **F** | **p** |
| **Intercept** | 2,931846 | 1 | 2,931846 | 793,1146 | 0,000000 |
| **Line** | 0,121919 | 10 | 0,012192 | 3,2981 | 0,003247 |
| **Error** | 0,151562 | 41 | 0,003697 |  |  |

**Figure 8.**

| Univariate Tests of Significance for Callose intensity measured as number of bright pixels (**Control, single mutant, double mutant lines**) Sigma-restricted parameterization Effective hypothesis decomposition | | | | | |
| --- | --- | --- | --- | --- | --- |
|  | **SS** | **Degr. of - Freedom** | **MS** | **F** | **p** |
| **Intercept** | 19341511 | 1 | 19341511 | 460,9962 | 0,000000 |
| **Line no** | 6364754 | 4 | 1591189 | 37,9253 | 0,000000 |
| **Error** | 629338 | 15 | 41956 |  |  |
